# Impact of changes in the methodology of external price referencing on medicine prices: discrete-event simulation

**DOI:** 10.1186/s12962-020-00247-3

**Published:** 2020-11-16

**Authors:** Sabine Vogler, Peter Schneider, Lena Lepuschütz

**Affiliations:** WHO Collaborating Centre for Pharmaceutical Pricing and Reimbursement Policies, Pharmacoeconomics Department, Gesundheit Österreich GmbH (GÖG, Austrian National Public Health Institute), Stubenring 6, A 1010, Vienna, Austria

**Keywords:** Pharmaceutical, Pricing policy, Methodology, Cost control

## Abstract

**Background:**

Several governments apply the policy of external price referencing (EPR), which considers the prices of a medicine in one or more other countries for the purpose of setting the price in the own country. Different methodological choices can be taken to design EPR. The study aimed to analyse whether, or not, and how changes in the methodology of EPR can impact medicine prices.

**Methods:**

The real-life EPR methodology as of Q1/2015 was surveyed in all European Union Member States (where applicable), Iceland, Norway and Switzerland through a questionnaire responded by national pricing authorities. Different scenarios were developed related to the parameters of the EPR methodology. Discrete-event simulations of fictitious prices in the 28 countries of the study that had EPR were run over 10 years. The continuation of the real-life EPR methodology in the countries as surveyed in 2015, without any change, served as base case.

**Results:**

In most scenarios, after 10 years, medicine prices in all or most surveyed countries were—sometimes considerably—lower than in the base case scenario. But in a few scenarios medicine prices increased in some countries. Consideration of discounts (an assumed 20% discount in five large economies and the mandatory discount in Germany, Greece and Ireland) and determining the reference price based on the lowest price in the country basket would result in higher price reductions (on average − 47.2% and − 34.2% compared to the base case). An adjustment of medicine price data of the reference countries by purchasing power parities would lead to higher prices in some more affluent countries (e.g. Switzerland, Norway) and lower prices in lower-income economies (Bulgaria, Romania, Hungary, Poland). Regular price revisions and changes in the basket of reference countries would also impact medicine prices, however to a lesser extent.

**Conclusions:**

EPR has some potential for cost-containment. Medicine prices could be decreased if certain parameters of the EPR methodology were changed. If public payers aim to apply EPR to keep medicine prices at more affordable levels, they are encouraged to explore the cost-containment potential of this policy by taking appropriate methodological choices in the EPR design.

## Background

Access to affordable medicines is a key policy objective in all countries of the world, and it has also been defined in the Sustainable Development Goals [1]. Medicine prices are one important determinant to ensure affordable and equitable access to medicines [2]. Affordable prices are of relevance both for patients who have to purchase medicines out-of-pocket or co-pay to the medicine price as well as for public payers (e.g. a social health insurance, a national health service) that cover (parts of) pharmaceutical expenditure. While the reduction in out-of-pocket payments for medicines lowers the risk for impoverishment of households [3–5], savings in public pharmaceutical budgets allow public payers to treat more patients and to contribute to the financial sustainability of the health care system [6].

To achieve affordable medicine prices, governments can employ a mix of pharmaceutical pricing policy options [7]. To set medicine prices, external price referencing (EPR) has increasingly been used. Applying this policy, the pricing authority or public payer considers the prices of a medicine in one or more other countries in order to derive a price benchmark for the purposes of setting or negotiating the price of a medicine in the own country [8]. Meanwhile, EPR has been implemented in many high-income countries and several middle-income countries [9–11]. Some countries without price regulation that aim to introduce price control have also opted for EPR as the primary pricing policy [12].

While, in principle, EPR can be applied for all kinds of medicines, it is, in practice, mainly used for pharmaceuticals with a new active substance that have no equivalent or therapeutically comparable medicine on the market. It is supplemented by further policies if public payers consider the EPR-based benchmark price unaffordable or unacceptable. In such cases, the payer and the pharmaceutical company tend to follow up by negotiating a lower price, which may, or may not, be linked to specific conditions (e.g. capping of the number of patients treated, price–volume agreements or clinical outcomes in pay-for-performance arrangements). In Europe, such arrangements are referred to as managed-entry agreements (MEA) [13]. The extent of discounts granted by industry to the payers, and thus the actual net prices are, as a rule, kept confidential in the MEA [14]. The non-disclosure of the discounts has an impact on other EPR-applying countries: Since legislation in nearly all EPR-applying countries provides for referencing to list prices, countries have to refer to the officially published, thus higher, list prices for their EPR calculations [15, 16].

Further methodological choices of EPR, however, vary across countries. For instance, in 2018, 18 of the 41 Pharmaceutical Pricing and Reimbursement Information (PPRI) countries^1^ that applied EPR used the average or median of the prices in other countries, while nine countries referred to the lowest prices and the remaining countries applied other algorithms to determine the benchmark prices. A basket of fewer than ten reference countries is used by 20 EPR-applying PPRI countries, whereas five PPRI countries reference to all or nearly all other EU Member States [12].

Overall, evidence on the impact of pharmaceutical pricing policies is still lacking [17, 18]. Some studies showed that the introduction and implementation of EPR has contributed to lower medicine prices and/or savings in public budgets [19–24]. Other research, however, pointed to the inferiority of EPR’s ability as cost-containment tool compared to other pricing policies, such as value-based pricing, or showed inconclusive results [25–28].

As for any other policy, the ability of EPR to achieve defined policy objectives, including cost-containment, largely also depends on its design, such as the number and the selection of reference countries and the chosen methodology to derive the benchmark price. Thus, it is key that policy-makers carefully decide on the methodological specifications of the EPR design, as this was also stressed by policy guidance documents [17, 29]. However, there is lack of information on the cost-containment potential of the different parameters that make up the EPR policy. Espin et al. [30] argued that modelling and scenario-building approaches could be appropriate tools to assess the impact of EPR.

Against this background, the paper aims to analyse the impact that the EPR methodology, in particular of its parameters, can have on medicine prices. The findings are intended to provide evidence to policy-makers who plan to introduce, or adapt, EPR.

## Methods

The study used a simulations model to investigate the development of medicine prices in EPR-applying countries over a period of 10 years for several scenarios in which EPR parameters were changed compared to the respective existing country methodologies.

### Discrete-event simulations

The model was structured as discrete-event simulations (DES). DES are an operations research modelling and analysis methodology which allows evaluating the efficiency of health care systems and policy measures, to ask ‘what if?’ questions and to design new system operations and policies. DES are also used as a forecasting tool to assess the potential impact of changes on defined indicators [31], such as done in this study, in which the impact of changes in the EPR methodology on medicines prices in the selected countries was explored.

### Included countries, their attributes and time horizon

The study included all European Union (EU) Member States whose legislation had implemented EPR for (at least some) outpatient medicines in the year 2015. These were 25 of the then 28 EU Member States: Austria, Belgium, Bulgaria, Croatia, Cyprus, Czech Republic, Estonia, Finland, France, Germany, Greece, Hungary, Ireland, Italy, Latvia, Lithuania, Luxembourg, Malta, the Netherlands, Poland, Portugal, Romania, Slovakia, Slovenia and Spain. Denmark, Sweden and United Kingdom were excluded since they did not apply EPR, or only in the hospital sector, as it was the case for Denmark. Additionally, three of the four European Free Trade Association (EFTA) member countries were included: Iceland, Norway and Switzerland; they all use EPR. The fourth EFTA country, Liechtenstein, was excluded because it applies the prices of Switzerland.

The simulated attributes were parameters which define the EPR methodology: the reference countries, the calculation of the benchmark price, consideration of discounts, weighting of the price data of the other countries and the frequency of revisions.

The DES model allows tracking of different agents (in the case of this study: countries), through a number of defined events. The simulations were run over a 10 years (120 months) horizon, with 1 month being taken as the basic time period. After an initial kick-off with two countries (Germany and Italy), the EPR-based prices were set in the other countries as soon as the minimum required number of price data in the reference countries was available. Thus, in the initial stage, no EPR benchmark price was available in some countries. In the follow-up phase, prices were held constant until a price revision was due according to legislation. No price deflation or inflation was considered. Exchange rates were assumed constant over time.

Based on the DES model, it was studied for each included country at every discrete point (i.e. on a monthly basis) whether, or not, its legislation provided for a revision, and if this were the case, the benchmark price was newly set. It was expected that possible changes in the prices in the reference countries might eventually also impact the price in the price-revising country.

The simulations were run using Stata 13.1.

### Assumptions and simulation scenarios

Simulations were run for fictitious prices (ex-factory price level).

To kick-off, a launch price of 100 euro was assumed for Germany and of 70 euro for Italy. Germany was selected for the kick-off since empirical evidence pointed to its status of first launch market in many cases [32, 33]). Italy qualified as the second starting country because it was referenced by several other countries and it had a large basket of reference countries [12]. In the other countries the EPR benchmark prices would only be determined after the prices in the defined minimum number of reference countries were available. If a country had no defined minimum number of reference countries in legislation, the simulations started as soon as price data were available in at least one country. If Denmark, Sweden or UK served as reference countries, their prices were assumed to amount to 100 euro and were held constant at this level throughout the 120 months.

Eight scenarios were run. The first one was the ‘base case’ scenario which considered for each included country the parameters of the EPR methodology as actually implemented in 2015 (Table 1). Seven further scenarios were developed, which were based on the assumption of a change in one parameter of the EPR methodology (Table 1). In the scenarios, the changed parameter was assumed to be applicable for all countries included in the simulations, while the other parameters did not change and were applied in the same way as in the base case scenario, thus they were country-specific and differed between the countries.

**Table 1:** Simulation scenarios—assumptions of changes in the EPR methodology

Scenario name	Simulated parameter	Assumed change in the design of the EPR methodology
Base case	–	For all included countries, the EPR methodology as in place in 2015 was considered. Included parameters: reference countries, the calculation method to determine the EPR benchmark price, the consideration of statutory discounts (i.e. outlined in legislation) and of commercially negotiated discounts, weighting of price data in other countries by volume data and/or by purchasing power parities, and the frequency of revisions of prices based on the review of the prices in the reference countries
Strategic basket	Reference countries	It was assumed that all included countries would have four reference countries: Germany, Italy, Finland and Portugal (this assumed country basket represented a mix of high-, middle- and low-priced countries)
Large basket	Reference countries	It was assumed that all included countries would have a basket of 30 reference countries (i.e. reference countries would be all other countries out of the group of the 28 EU Member States and the three EFTA countries Norway, Iceland and Switzerland)
Lowest price	Calculation method	It was assumed that all included countries would reference to the reference country with the lowest price
Statutory discounts	Discounts	It was assumed that all included countries would consider the statutory manufacturer discounts in Germany, Greece and Ireland (the amount of statutory discounts that pharmaceutical companies have to grant the public payers are outlined and published in legislation in Germany and Greece and in a framework agreement in Ireland)
Statutory and commercial discounts	Discounts	It was assumed that all included countries would consider the statutory manufacturer discounts in Germany, Greece and Ireland and an additional assumed 20% (confidential) discount on the referenced prices in five large economies (Germany, France, Italy, the Netherlands, Spain and UK)
PPP	Income adjustment	It was assumed that all included countries would weight the price data of the reference countries by Purchasing Power Parities (PPP)
Bi-annual revisions	Revision frequency	It was assumed that all included countries would review the price data of the reference countries every 6 months and would subsequently adjust appropriately the medicine prices in the own country

### Survey of existing EPR systems

The parameters of existing EPR systems as in place in the analysed countries in 2015, which served as input for the attributes of the base case scenario, were surveyed with competent authorities for pharmaceutical pricing and reimbursement. Based on literature and previous primary data collections (as part of information sharing activities in the PPRI network of competent authorities [34]), the authors drafted for all included countries a description of the EPR design, which specified all analysed parameters. The authorities were addressed in the first quarter of 2015 to review and validate the information for their country.

## Results

### Real-life EPR methodology

The survey of the EPR methodology applied in the studied countries had a 100% response rate following persistent reminders. Variation in the EPR methodology was observed particularly related to the number of reference countries: While Luxembourg considered the prices of solely one country, Hungary and Poland had 31 countries in the basket. In most surveyed countries the basket included between eight and 15 countries. In some countries (e.g. Italy, Spain) the number of reference countries was not explicitly defined but a larger group of countries (e.g. those from the Euripid database or the Euro zone) served as reference. Differences were also found with regard to the method to determine the reference price: common methodologies included the average and the minimum of the prices in the reference countries, but a combination, or a slightly different calculation method was also applied in a few countries (for instance, Latvian medicine prices should be third lowest of the basket of seven reference countries but they should not exceed the price in Lithuania or Estonia). Several EPR-applying countries reviewed their prices bi-annually and annually, but some countries have not provided for any revision in legislation. Except for Germany, none of the countries had in 2015 a legislation in place to take into account discounts (not even published mandatory discounts) or to weight price data of other countries (Table 2).

**Table 2:** Parameters of the EPR methodology in the studied EPR-applying countries, 2015

Country	Reference countries^a^	Benchmark price	Consideration of discounts	Weighting of price data	Revision frequency (months)
Austria	26 [14]	Average	No	No	No revision
Belgium	27 [1]	Average	No	No	No revision
Bulgaria	17 [1]	Minimum	No	No	6
Croatia	3 out of 5^b^ [2]	Average	No	No	12
Cyprus	4 out of 10^b^ [1]	Average	No	No	12
Czech Republic	19 [3]	Average of 3 lowest	No	No	36
Estonia	3 [1]	Minimum	No	No	12
Finland	29 [1]	Average	No	No	60
France	4 [1]	Average	No	No	60
Germany	15 [1]	Average	Provided for in legislation^c^	Provided for in legislation^c^	No revision
Greece	26 [3]	Average of 3 lowest	No	No	3
Hungary	31 [3]	Minimum	No	No	No revision
Iceland	4 [3]	Average	No	No	24
Ireland	9 [1]	Average	No	No	36
Italy	25^d^ [1]	Minimum	No	No	24
Latvia	7 [1]	Third lowest price	No	No	24
Lithuania	8 [1]	Average	No	No	12
Luxembourg	1 [1]	Minimum	No	No	12
Malta	12 [3]	Average	No	No	18
Netherlands	4 [2]	Average	No	No	6
Norway	9 [1]	Average of 3 lowest	No	No	12
Poland	31 [1]	Average	No	No	24
Portugal	3 [1]	Average	No	No	12
Romania	12 [1]	Minimum	No	No	60
Slovakia	27 [1]	Average of 3 lowest	No	No	6
Slovenia	3 [1]	Minimum	No	No	6
Spain	18^e^ [1]	Minimum	No	No	12
Switzerland	6 [1]	Average	No	No	36

^a^In bracket the number of minimum reference countries that are required in legislation to determine an EPR benchmark price

^b^3 (Croatia) and 4 (Cyprus) defined reference countries, respectively, out of a pool of 5 (Croatia) and 10 (Cyprus) reference countries, as data of alternative reference countries are considered in the case of non-availability of data in the primary 3 or 4 reference countries

^c^According to legislation, Germany can consider mandatory and confidential discounts of prices in other countries, but this is not applied in practice. Furthermore, Germany has the legal mandate to weight the price data by estimated yearly turnover of the medicine (information to be provided by the pharmaceutical company) and by purchasing power parities (PPP). As both discounts as well as weighting are no common practice in the EPR in Germany, this was not considered in the base case simulations

^d^Countries with price data included in the Euripid database

^e^Eurozone countries

### Simulations

**Fig. 1:**
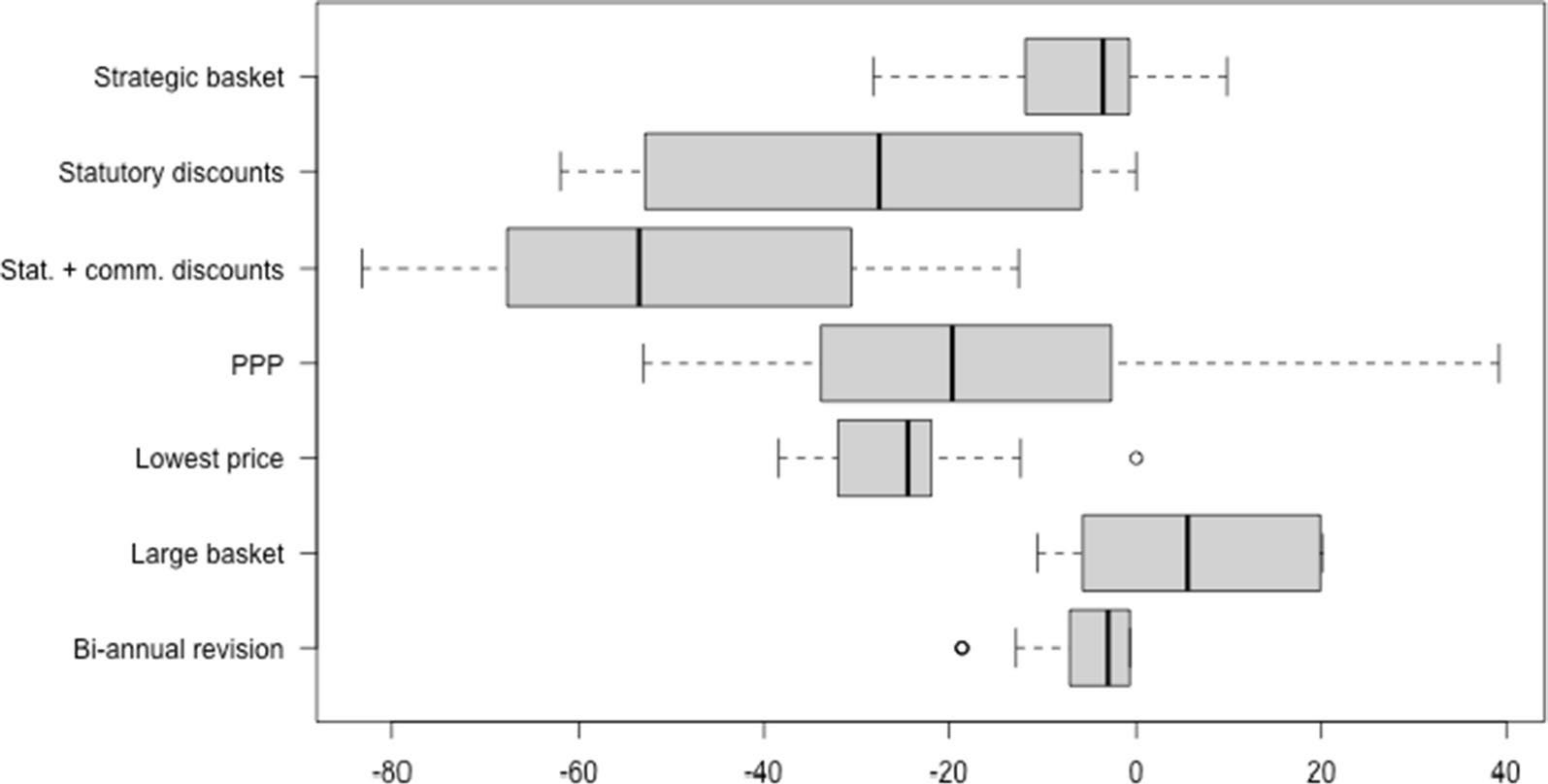
Boxplot on the change in medicine prices after 10 years, upon modification in one of the parameters of the EPR methodology, compared to the base case (‘no change’)

While in some scenarios after 10 years medicine prices in all or most surveyed countries were—sometimes considerably—lower than in the base case (i.e. if the countries continued applying their existing EPR methodology without any change), medicine prices also increased in some countries in a few scenarios (Fig. 1 and Additional file 1: Table S1).

Out of all scenarios, consideration of discounted prices had the highest impact, in particular when a 20% discount was assumed for five large economies in addition to the consideration of statutory discounts: in this case (scenario ‘statutory and commercial discounts’), the prices were, on average, 47.2% lower than the prices achieved through the application of the existing EPR methodology. When solely statutory discounts were considered, the average reduction amounted to 26.8% compared to the prices in the base case scenario, with higher reductions of up to 62% in some countries (Bulgaria, Greece, Italy, Romania, Slovakia and Spain). Simulated prices in the scenario with statutory and commercial discounts were on average 50% lower in the majority of the countries (over 80% in Croatia and Lithuania).

A considerable effect was also shown in the case of a change in the calculation methodology when all analysed countries referenced to the reference country with the lowest price: on average 34.2% lower prices than in the base case scenario.

Bi-annual price reviews would also lead to lower prices on average and in all studied EPR-applying countries, but to a lower extent (less than 1 per cent in several countries).

Weighting price data of the reference countries by PPP showed mixed results: medicine prices of lower-income countries would be reduced, while the adjustments would lead to higher prices in high-income countries. In particular, prices in Switzerland and Norway would increase by nearly 40% and 18% respectively, whereas Bulgarian, Romanian and Hungarian prices would decrease by 53%, 51% and 49% respectively. After 10 years, PPP-adjusted prices would be, on average, 16% lower than base case prices.

Findings differed with regard to the assumed reference countries: whereas a low number of strategically selected reference countries resulted, in general, in lower medicine prices (except for a few countries), a large basket led to higher prices compared to the base case in several countries.

## Discussion

The results strongly suggest that the methodological design of the EPR policy has an impact on the intended outcomes, i.e. the medicine prices in the own country. In particular, the simulations run in this study point to considerable relevance of some of the parameters of the EPR methodology. Thus, the findings add to studies that suggested EPR’s ability to reduce medicine prices [19–24].

It has been highlighted in literature [28, 35–37] and in policy debate that the cost-containment capacity of EPR is strongly impaired by referencing to ‘fake prices’ [38] since the real prices are not known due to the confidential character of discounts and managed-entry agreements. Both scenarios of this study that considered discounts confirmed the loss of savings opportunities given the non-consideration of discounted prices in other countries. Even the scenario that only took into account statutory (thus published) discounts showed important price-reducing potentials. As the consideration of published discounts would not imply any breach of confidentiality, an EPR-applying country could implement it rather swiftly. In fact, since its medicine pricing reform of 2017 price data reduced by statutory discounts are taken into consideration in Austria [39]. Another technical option for governments to account for discounts could be to follow the example of scenario ‘statutory and commercial discounts’ and to assume a ‘reasonable’ discount. At political level, a debate on price transparency is ongoing, as evidenced by the ‘WHO Transparency Resolution’ adopted by the World Health Assembly in May 2019. This WHO Resolution calls for disclosure of net prices as well as research and development costs for medicines and vaccines [40].

It can be expected that the approach to determine the EPR benchmark price by referring to the lowest priced reference country will lead to lower prices compared to other methodologies. This was, not surprisingly, confirmed by the simulations results. The base case scenario included some European countries that calculated their reference price based on the average of the prices in the reference countries, and particularly for these countries, major decreases compared to the base case were seen. However, it can be discussed whether, or not, a policy of a ‘race to the bottom’ is an intended objective of EPR that is a pricing policy usually applied for new medicines. As an alternative, opportunities for savings could also be achieved from off-patent medicines, as evidence on the price-reducing character of generic competition [41–45] and of tendering [46–50] is available.

The selection of the reference countries is a key decision point in the design of EPR. The WHO Guideline on Country Pharmaceutical Pricing Policies recommends choosing reference countries based on a set of explicitly stated factors [17]. With regard to the reference countries, policy-makers have to take two major choices: the size of the basket and the countries to include. With regard to the latter, it is common sense that a focus on lower-priced reference countries will eventually lead to lower prices. There is, however, the risk that particularly in the beginning price setting might be difficult because medicines might not have a price and be marketed in lower-priced countries due to strategic launches of pharmaceutical companies in response to the widespread use of EPR [32, 51, 52]. Thus, countries, particularly those referring to lower-priced countries, are advised to have a mechanism in place which allows setting the price even with the product being marketed in a few countries (e.g. alternative countries) and provides for regular price reviews to benefit from price decreases in the reference countries over the years (see also findings of scenario ‘bi-annual revisions’) [18]. As a related aspect, it has to be decided whether, or not, there is a need to have large country baskets. This has also to be seen in connection with the resources required for surveying medicine price data to perform EPR, which can be substantial in case of large country baskets [53]. In any case, the study findings confirm the importance of a strategic selection of the reference countries: a well-chosen small country basket is not only less resource-intensive but may also achieve lower prices. Indeed, the simulations showed that most countries would pay higher prices (increases by 20% in several cases) if they used a larger basket (scenario ‘large basket’ with the assumption of 30 reference countries).

EPR has been criticised for failing to deliver equity since it does not consider the different income levels of the reference countries [27, 54]. As a solution, differential pricing—a policy in which medicine prices are set in line with the countries’ economic status—has been proposed [55–58]. Usually, differential pricing and EPR are considered as mutually exclusive policy options. However, in the authors’ perception, this is not necessarily the case. For instance, the prices in the reference countries could be weighted by indicators that reflect the economic situation of these countries (e.g. gross domestic product, PPP). The simulation scenario that was run on PPP-adjusted prices showed lower prices for lower-income countries but also an increased burden due to higher prices for higher-income countries. While accounting for countries’ income would contribute to more equity and fairness, such an approach may still be politically acceptable for high-income countries that are meanwhile also struggling to afford medicines.

The authors acknowledge that the study has limitations. The simulations model had to be based on assumptions (e.g. on the starting price and the kick-off countries), which were simplified compared to the far more complex reality. It was decided to focus on pricing of medicines in the outpatient sector, which resulted in the exclusion of Denmark (EPR only applicable for some hospital medicines). EPR was assumed to be the sole pricing policy in the EPR-applying countries. In the studied countries, the EPR policy is not always used for all medicines but other pricing policies are also applied. For instance, internal price referencing, which considers the prices of comparable medicines (e.g. of the same active ingredient) in the same country, is also commonly applied, in particular for medicines in the off-patent market (e.g. generics, biosimilars, and originator medicines whose patent has expired). Even if EPR is applied, some countries accompany its use by further policies; e.g. price negotiations, for which the EPR-determined benchmark price rather serves as background information.

No scenario in which the price data were weighted by volume was run as these data were not available. Governments frequently lack consumption data of other countries. Weighting by volume data of the own country could be applied as a back-up option, but even these data are not always accessible (e.g. in countries with a fragmented health care system, such as Austria and Germany, aggregated consumption data for the hospital sector are missing).

Medicine prices were the sole outcome indicator of the study. Pharmaceutical prices are indeed a major contributing factor for affordable and equitable access to medicines [2] because in solidarity-based systems (as those of the studied countries) lower prices allow the public payers to treat more patients for the same amount spent. But pharmaceutical expenditure is also influenced by volume. Thus, even if prices were decreased, expenditure may grow as a result of increases in consumption [19]. The latter may be attributable to over-use or inappropriate use and also to adjustments of previous under-use. Furthermore, public pharmaceutical expenditure may be lower since the published list prices based on EPR are not the reimbursement prices; confidential discounts reduce the price that the public payers actually pay.

Analysing medicine prices as outcome parameter, the study focused on the cost-containment potential of EPR. Thus, it did not consider further objectives that policy-makers may aim to achieve, e.g. to facilitate timely access to medicines, to support the local industry, to incentivise research-oriented pharmaceutical industry to invest into research and development or to ensure that the same price for a medicine is charged in all pharmacies throughout the whole country. Other pharmaceutical (pricing) policies might be more appropriate to reach these objectives.

Finally, the study only analysed the impact of the EPR methodology on the prices in the same countries. EPR is known for its spill-over effects on access in other countries (e.g. launch delays in lower-income countries with lower medicine prices) [11, 51, 52] but this was not scope of this research.

Despite its limitations, the research has important policy implications: It reminds policy-makers to carefully take methodological choices when they implement EPR. It is not simply a question of whether, or not, they apply EPR, but also how they do so. Some parameters of the EPR methodology generate higher cost-containment impacts than others, and if pricing authorities and public payers aim to achieve lower prices through EPR, specific parameters, i.e. consideration of discounts and lowest prices as benchmark, appear to be most appropriate ones. The first, however, would imply a major change in the design of most EPR policies in place, since, as shown in the survey, only very few countries consider mandatory discounts which are publicly accessible. Changing EPR legislation by indicating discounted prices as reference would additionally signal the interest of governments to overcome the current challenge of ‘fake prices’ [38].

## Conclusions

The EPR policy is one of the most commonly applied pharmaceutical pricing policies, with the aim to achieve affordable and sustainable medicine prices. While the study neither advocates in favour nor against the use of EPR, its findings have shown the ability of this policy to contribute to lower medicine prices in general and in particular in the case of specific methodological choices. Consideration of discounts and the application of the lowest price formula (instead of the average of prices) in the reference countries for the calculation of the benchmark price represent parameters with the highest potential to decrease medicine prices.

The study points to the importance of a careful design of the EPR policy. Thus, policy-makers are recommended to take appropriate methodological choices and to detail in legislation the specifications of the EPR design when they introduce this policy. Given its high dependency on the developments in the other countries that serve as reference, EPR should be evaluated regularly with a view to explore whether, or not, the chosen methodology continues achieving the intended objectives. If needed, the methodology should be adjusted.

## Supplementary information

**Additional file 1: Table S1.** Change in medicine prices after 10 years, upon modification in one of the parameters of the EPR methodology, compared to the base case (‘no change’).

## Data Availability

All data generated or analysed during this study are included in this published article.

## Abbreviations

EFTA: European Free Trade Association
EPR: External price referencing
EU: European Union
HTA: Health technology assessment
MEA: Managed-entry agreement
PPP: Purchasing Power Parities
PPRI: Pharmaceutical Pricing and Reimbursement Information
UK: United Kingdom
WHO: World Health Organization

## Footnotes

1 PPRI is a network of pharmaceutical pricing and reimbursement authorities in 47 countries (as of 2018), including all 28 Member States of the European Union and further countries in Europe (e.g. in the Balkans) and Central Asia as well as Canada, Israel, South Africa and South Korea.
