# Supplementary material for: Impact of changes in the methodology of external price referencing on medicine prices: discrete-event simulation
Source: Cost Eff Resour Alloc. 2020 Nov 16;18:51. doi: 10.1186/s12962-020-00247-3 (PMC7670789; doi:10.1186/s12962-020-00247-3)
Supplement: Supplementary file 1 — Additional file 1: Table S1. Change in medicine prices after 10 years, upon modification in one of the parameters of the EPR methodology, compared to the base case (‘no change’). [file 12962_2020_247_MOESM1_ESM.docx]

**Table S1.** Change in medicine prices after ten years, upon modification in one of the parameters of the EPR methodology, compared to the base case (‘no change’)

| **Parameter with change** | ***No change*** | **Reference countries** | | **Calculation method** | **Discounts** | | **Income adjustment** | **Revision frequency** |
| --- | --- | --- | --- | --- | --- | --- | --- | --- |
| **Scenario** | ***Base case*** | **Strategic basket** | **Large basket** | **Lowest price** | **Statutory** | **Statutory and commercial** | **PPP** | **Bi-annual revision** |
| Austria | *94.0* | 0.0% | -9.6% | **-25.5%** | -1.5% | -12.8% | -6.2% | -18.6% |
| Belgium | *94.0* | 0.0% | -9.6% | **-25.5%** | -1.5% | -12.8% | -5.4% | -18.6% |
| Bulgaria | *70.0* | -3.6% | 19.9% | -21.9% | **-59.0%** | **-72.9%** | **-53.0%** | -0.7% |
| Croatia | *67.8* | 4.0% | 13.6% | **-26.1%** | **-40.9%** | **-83.2%** | **-39.4%** | -3.1% |
| Cyprus | *74.9* | -10.5% | -2.2% | **-35.1%** | -15.3% | **-36.5%** | -22.2% | -5.4% |
| Czech Rep. | *70.0* | -3.1% | 19.9% | -19.7% | **-52.6%** | **-50.0%** | **-35.0%** | -0.7% |
| Estonia | *70.0* | -3.1% | 19.9% | -20.0% | **-53.1%** | **-54.3%** | **-27.7%** | -0.7% |
| Finland | *78.6* | -5.1% | 3.7% | **-33.0%** | -23.5% | **-52.7%** | 10.7% | -5.0% |
| France | *82.2* | -6.4% | 2.4% | **-33.5%** | **-27.1%** | **-58.0%** | 0.7% | -3.0% |
| Germany | *89.0* | -1.5% | -4.5% | -12.5% | -1.8% | -19.1% | -10.1% | -10.2% |
| Greece | *70.0* | -3.6% | 19.9% | -21.9% | **-58.3%** | **-68.6%** | -17.1% | -0.7% |
| Hungary | *70.0* | 0.0% | 0.0% | 0.0% | 0.0% | -20.0% | **-49.1%** | -0.7% |
| Iceland | *88.8* | -17.1% | -9.2% | **-38.4%** | -4.4% | -18.0% | 3.5% | -4.5% |
| Ireland | *89.1* | -13.5% | -5.5% | **-35.7%** | -9.4% | **-33.6%** | -7.0% | -7.4% |
| Italy | *70.0* | -3.6% | 19.9% | -21.9% | **-59.0%** | **-66.9%** | 0.0% | -0.7% |
| Latvia | *70.0* | -3.6% | 19.9% | -21.9% | **-52.6%** | **-50.7%** | **-32.7%** | -0.7% |
| Lithuania | *70.1* | 9.8% | 20.1% | -20.4% | **-40.8%** | **-80.5%** | **-39.8%** | -0.9% |
| Luxembourg | *94.0* | **-28.2%** | -10.7% | **-25.5%** | -1.5% | -12.7% | 1.8% | -18.6% |
| Malta | *75.5* | 2.1% | 11.5% | **-25.6%** | **-29.7%** | **-66.6%** | **-26.6%** | -2.0% |
| Netherlands | *81.6* | -15.7% | -7.7% | **-31.0%** | -6.5% | **-32.1%** | -8.0% | -8.0% |
| Norway | *84.0* | -24.2% | -6.1% | **-35.8%** | -9.2% | **-39.9%** | 18.3% | -13.0% |
| Poland | *70.6* | 8.9% | 19.3% | -22.5% | **-38.2%** | **-76.8%** | **-42.5%** | -1.1% |
| Portugal | *78.2* | -1.5% | 7.4% | **-28.6%** | **-28.0%** | **-71.6%** | **-28.3%** | -3.8% |
| Romania | *70.0* | -3.6% | 19.9% | -21.9% | **-62.0%** | **-73.6%** | **-50.9%** | -0.7% |
| Slovakia | *70.0* | -3.6% | 19.9% | -22.4% | **-58.0%** | **-65.3%** | **-32.7%** | -0.7% |
| Slovenia | *82.2* | -17.9% | 2.1% | -23.4% | -15.5% | **-60.9%** | **-28.3%** | -6.9% |
| Spain | *70.0* | -3.6% | 19.9% | -20.3% | **-57.6%** | **-58.6%** | -10.7% | -0.7% |
| Switzerland | *93.0* | -17.1% | -9.5% | **-33.0%** | -5.4% | **-29.0%** | **39.2%** | -7.0% |
| *Minimum* | *-* | ***-28.2%*** | *-10.7%* | ***-38.4%*** | ***-62.0%*** | ***-83.2%*** | ***-53.0%*** | *-18.6%* |
| *Average* | *78.1* | *- 6.5%* | *5.3%* | ***- 34.2%*** | ***- 26.8%*** | ***-47.2%*** | *- 16.0%* | *- 5.8%* |
| *Median* | *-* | *-3.6%* | *5.6%* | *-24.7%* | *-27.6%* | ***-53.5%*** | *-19.7%* | *-3.1%* |
| *SD* | *-* | *9.1* | *12.4* | *7.9* | *22.9* | *22.8* | *22.4* | *5.8* |
| *Max* | *-* | *9.8%* | *20.1%* | *0.0%* | *0.0%* | *-12.7%* | ***39.2%*** | *-0.7%* |

Rep. = Republic

The base case scenario shows the average medicine prices after 10 years if the country-specific parameters of the EPR methodology were continued to be applied over the years. The assumptions of the scenarios are described in Table 1.

Changes (decreases / increases) exceeding 25% are marked in bold.
